# A retrospective study of 43 cases of fungal malignant external otitis

**DOI:** 10.11604/pamj.2022.41.287.29585

**Published:** 2022-04-08

**Authors:** Chiraz Halwani, Latifa Mtibaa, Moez El Hamdi, Nawel Baccouchi, Rania Benmhamed, Boutheina Jemli, Khemaies Akkari

**Affiliations:** 1 Faculty of Medicine of Tunis, Tunis El Manar University, Tunis, Tunisia,; 2 Department of Otolaryngology (ENT), Military Hospital of Tunis, 1008 Monfleury, Tunis, Tunisia,; 3 Laboratory of Parasitology-Mycology, Military Hospital of Tunis, 1008 Monfleury, Tunis, Tunisia

**Keywords:** Malignant, fungal otitis, Candida, Aspergillus, Geotrichum

## Abstract

Malignant external otitis (MEO) has a frequent bacterial origin, but we are currently witnessing the emergence of fungal agents, which poses difficulties in diagnosis and management. The aim of our work is to analyze the epidemiological and clinical profile of fungal MEO and to study the antifungal susceptibility of fungi involved. Our study is retrospective collecting 43 patients treated for fungal MEO between 2010 and 2019. Clinical, biological, and radiological data were collected from patient hospitalization records. Identification of yeasts was done by YST vitek^®^2 card. The antifungal susceptibility testing was performed for yeasts by the AST vitek^®^2 card and for other fungi by the E-test technique. The average age was 66 (± 12) years. We noted a male predominance in 63 % (n=27). Diabetes was found in 86%. Otalgia was a constant symptom. Cranial nerve palsies were observed in 16% (n=7) of cases. CT showed bone lysis in 74% (n=31>) of cases and Tc99 bone scintigraphy revealed hyperfixation in 100% (n=43) of cases. Candida spp. (n=21), Aspergillus spp. (n=18), and Geotrichum capitatum (n=2) were isolated. No resistance to antifungals has been demonstrated for Candida yeasts. Geotrichum capitatum isolates were resistant to fluconazole and caspofungin. Aspergillus isolates were resistant to amphotericin B and caspofungin in 50% (n=9) and 72% (n=12) of cases, respectively. Our study proves the predominance of Candida yeasts and Aspergillus as the fungal agents involved in MOE. Mycological diagnosis allows the identification and antifungal susceptibility testing. Thus, it allows using of the appropriate antifungal treatment and improves the prognosis of the disease.

## Introduction

Malignant external otitis (MEO) corresponds to osteomyelitis of the skull base starting in the external auditory canal [1]. It is a serious and rare infectious emergency with an overall mortality of around 20% [1]. Its severity is due to the risk of the extension of the infection to the central nervous system or to the deep spaces of the face and its occurrence mostly in elderly diabetic, immunocompromised subjects. It has frequently bacterial origin, but we are currently witnessing the emergence of fungal agents, which poses difficulties in diagnosis and management. *Candida* yeasts and *Aspergillus* are the fungal pathogens mainly incriminated [2,3]. The majority of studies found in the literature are case reports or small series. The aim of our work is to analyze the epidemiological and clinical profile of this infection and to study the antifungal susceptibility of fungi involved.

## Methods

**Study design and setting**: our study is retrospective collecting 43 patients treated for MEO at the Tunis Military Hospital over a period of 10 years (January 2010 to December 2019).

**Study population**: this study included all patients who had been treated in the ENT department for MEO, in whom the fungus was identified on a mycological sample and/or on a positive *Aspergillus* antigenemia. Patients with unusable medical records were excluded from the study, in particular when the course under treatment was not well detailed or was lost to follow-up. The ear sample was taken by swabbing the external auditory canal under otoscopic examination. The identification of the yeasts was carried out using the YST vitek^®^2 card (Biomérieux). The identification of *Aspergillus* relied on their macroscopic and microscopic characters. The antifungal susceptibility testing was performed for yeasts by the AST vitek^®^2 (Biomérieux) card and for Aspergillus and *Geotrichum capitatum*, by the E-test technique with reference to the EUCAST recommendations. *Aspergillus* serology and antigenemia were performed using the PLATELIA^™^ *Aspergillus Ig*G (Biorad) and PLATELIA^™^Aspergillus Ag (Biorad) respectively.

**Data collection**: clinical, biological and radiological data were collected from patient hospitalization records and laboratory records.

**Statistical analysis**: data were entered using Excel software and analyzed using SPSS version 20 software. We calculated simple frequencies and relative frequencies (percentages) for the categorical variables. We calculated averages; medians and standard deviations and determined the extreme values (minimum and maximum) for the quantitative variables.

**Ethical consideration**: the global rules of ethics relating to respect for confidentiality and the protection of patient-specific data were taken into account during this work. We obtained the informed consent from patients and the authorization of the Ethical Committee of the Military Hospital of Tunis.

## Results

**Sociodemographic characteristics**: the diagnosis of MEO was confirmed in 43 patients, including 41 cases with mycological confirmation and 2 cases through positive antigenemia. The average age of our patients was 66 (± 12) years with extremes from 37 to 89 years. The proportion of males was 63 % (n=27). Diabetes was found in 86% (n=37).

**Clinical features**: otalgia was the main reason for consultation (100%, n=43) followed by otorrhea (81%, n=35). Cranial palsies were observed in 16% (n=7) of cases. Computed tomography of the rocks was performed in all patients and showed bone lysis in 74% (n=31) of cases (Table 1). Technetium-99 bone scintigraphy was performed for 37 patients (86%, n=37) and showed bone hyperfixation in all cases. Table 1 summarizes clinical, biological and radiological data of patients.

**Table 1:** clinical, biological and radiological characteristics of patients with fungal malignant external otitis

Variables	Number	Percentage
**Sex**		
Male	27	63%
Female	16	37%
**Age**		
<40 ans	1	2%
40-60 ans	9	21%
>60 ans	33	77%
**Antecedents**		
Diabetes	37	86%
Chronic renal failure	3	7%
Corticosteroid therapy	2	5%
Chemotherapy	1	2%
**Symptoms**		
Otalgia	43	100%
Otorrhea	35	81%
Hearing loss	20	46%
Facial asymmetry	11	26%
Tinnitus	8	18%
Fear of heights	4	9%
Trismus	2	5%
Fever	2	5%
**Extension**		
VII paralysis	6	14%
lesion of X, IX and XII	1	2%
Inflammation of the preauricular region	6	14%
Temporomandibular arthritis	8	19%
Collection	3	7%
Peri-chondritis	1	2%
**Sedimentation Rate**		
<20 mm/h	3	7%
20-40 mm/h	5	12%
>40 mm/h	35	81%
**HBA1C**		
≤7%	6	14%
7.1-10%	13	30%
>10%	24	56%
**CT scan**		
Bone lysis	32	74%
Filling of the external auditory canal	14	32%
Soft tissue collection	3	7%
Thrombosis of the lateral sinus or jugular vein	2	5%

**Figure 1:**
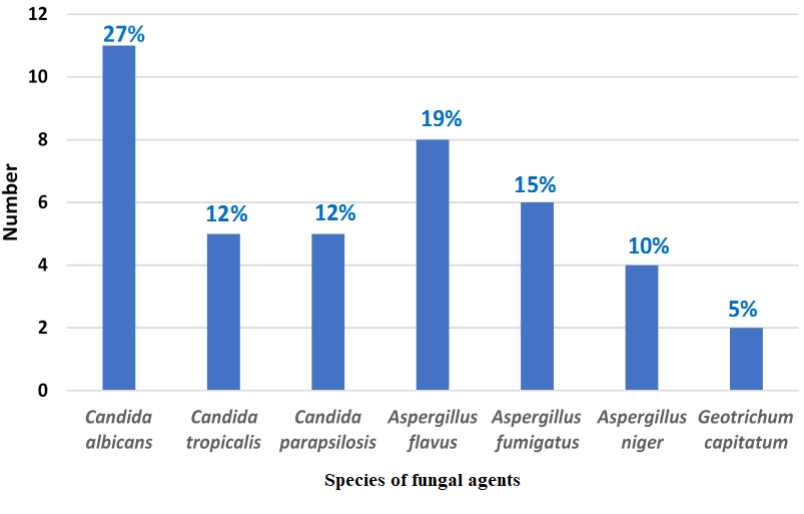
species of fungal agents isolated in culture

**Figure 2:**
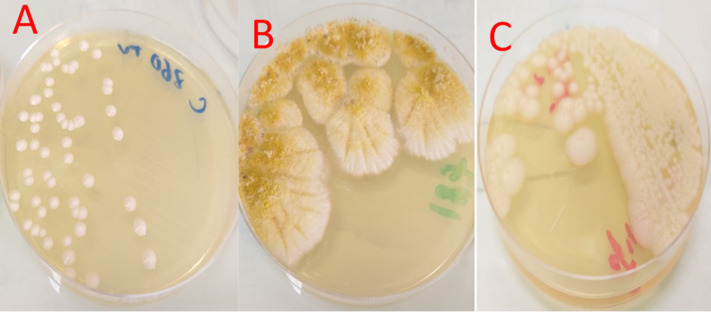
macroscopic aspect of cultures on sabouraud medium, A) *Candida ablicans*; B) *Aspergillus flavus*; C) *Geotrichum capitatum*

**Mycologic features**: the isolated fungi were: yeasts genus *Candida* (n=21), *Aspergillus* (n=18) and *Geotrichum capitatum* (n=2). *Candida albicans* was the most implicated strain in 11 patients (27%, n=11) followed by *Aspergillus flavus* in 8 patients (19%, n=8) and *Aspergillus fumigatus* in 6 patients (15%, n=6) (Figure 1). The three genera of isolated fungi are photographed and illustrated in Figure 2. Serology and antigenemia were performed in 18 cases. Serology was positive in 10 patients (55%, n=10). *Aspergillus* antigenemia was positive in only 2 patients (11%, n=2) having negative mycological cultures. No resistance to antifungals was demonstrated for yeasts of the *Candida* genus. All *Candida* yeasts were sensitive to Fluconazole and Voriconazole. *C. parapsilosis* isolates (n=5) were intermediate to Micafungin in 80% of cases (n=4). The results of the susceptibility of *Candida* yeasts to antifungals are shown in Table 2.

**Table 2:** antifungal sensitivity of *Candida* yeasts

Species/ Antifungal	*Candida albicans* (n=11)		*Candida parapsilosis* (n=5)		*Candida tropicalis* (n=5)	
	S	I	S	I	S	I
AMB	(100%)	(0%)	(100%)	(0%)	(100%)	(0%)
FLU	(100%)	(0%)	(100%)	(0%)	(100%)	(0%)
VCZ	(100%)	(0%)	(100%)	(0%)	(100%)	(0%)
CAS	(100%)	(0%)	(100%)	(0%)	(100%)	(0%)
MIC	(100%)	(0%)	(20%)	(80%)	(100%)	(0%)
5FC	(100%)	(0%)	(100%)	(0%)	(100%)	(0%)

**AMB:** Amphotericin B; **FLU:** Fluconazole; **VCZ**: Voriconazole; **CAS**: Caspofungin; **MIC**: Micafungin; **5FC** : 5 Fluorocytosine; **S**: Susceptible; **I**: Intermediate

For the genera *Aspergillus* and *Geotrichum*, Antifungal susceptibility was interpreted referring to EUCAST recommandations. The MICs found for all isolates are shown in Table 3. The two isolates of *Geotrichum capitatum* had low MICs for amphotericin B and voriconazole (<1µg/ml) and were resistant to fluconazole and caspofungin. *Aspergillus* isolates were all susceptible to voriconazole. Resistance to amphotericin B and caspofungin was reported in 50% (n=9) and 72% (n=13) of cases, respectively. Antifungal treatment consisted of fluconazole (49%, n=21) and voriconazole (51%, n=22) in case of MEO due to *Candida* yeasts and to *Geotrichum/Aspergillus* respectively. The duration of antifungal treatment varied between 3 and 7 months, with an average of 90 days. Other therapies were used such as surgery and hyperbaric oxygen therapy performed in 5 cases (12%) and 12 cases (28%) respectively. The evolution of our patients under treatment was favorable in 39 cases (91%, n=39) with a total cure of the disease without recurrence with an average follow-up of 2 years.

**Table 3:** in vitro sensitivity of *Geotrichum capitatum* and *Aspergillus* by the E-test technique

Variables	MICs (sensitivity)			
Fungal species (number of isolate)	Amphotericin B (µg ml-1)	Voriconazole (µg ml-1)	Fluconazole (µg ml-1)	*Caspofungine* (µg ml-1)
***Geotrichum capitatum* (1)**	0.38	0.25	**6**	**>32 (R)**
***Geotrichum capitatum* (2)**	0.5	0.19	**8**	**>32 (R)**
***Aspergillus flavus* (1)**	**>32 (R)**	0.064	**-**	**>32 (R)**
***Aspergillus flavus* (2)**	**>32 (R)**	0.25	**-**	**>32 (R)**
***Aspergillus flavus* (3)**	**>32 (R)**	0.064	**-**	**>32 (R)**
***Aspergillus flavus* (4)**	**>32 (R)**	0.125	**-**	0.25
***Aspergillus flavus* (5)**	**>32 (R)**	0.094	**-**	0.38
***Aspergillus flavus* (6)**	**>32 (R)**	0.125	**-**	0.25
***Aspergillus flavus* (7)**	**>32 (R)**	0.064	**-**	**>32 (R)**
***Aspergillus flavus* (8)**	0.38	0.125	**-**	**>3 (R)**
***Aspergillus fumigatus* (1)**	16 **(R)**	0.125 (S)	-	**>32 (R)**
***Aspergillus fumigatus* (2)**	0.75 (S)	0.047 (S)	-	**>32 (R)**
***Aspergillus fumigatus* (3)**	1 (S)	0.125 (S)	-	**>32 (R)**
***Aspergillus fumigatus* (4)**	0.75 (S)	0.125 (S)	-	**>32 (R)**
***Aspergillus fumigatus* (5)**	1 (S)	0.125 (S)	-	**>32 (R)**
***Aspergillus fumigatus* (6)**	0.75 (S)	0.125 (S)	-	**>32 (R)**
***Aspergillus niger* (1)**	0.75 (S)	0.125 (S)	-	**>32 (R)**
***Aspergillus niger* (2)**	**>32 (R)**	0.125 (S)	-	**>32 (R)**
***Aspergillus niger* (3)**	0.25 (S)	0.38 (S)	-	0.38
***Aspergillus niger* (4)**	0.75 (S)	0.064 (S)	-	0.38

## Discussion

In our study, fungal MEO diagnosis was confirmed in 41 cases with mycological examination and in 2 cases with positive Aspergillus antigenemia. *Candida spp*. (n=21), *Aspergillus spp*. (n=18), and *Geotrichum capitatum* (n=2) were isolated. All *Candida* yeasts were sensitive to fluconazole and voriconazole. Both isolates of *Geotrichum capitatum* had low MICs for amphotericin B and voriconazole. The *Aspergillus* isolates were all sensitive to voriconazole. Fungal MEO is a potentially serious and fatal disease that typically affects older people. The infection is favored by an imbalance of local and general defense mechanisms, in addition to virulence factors of fungal agents [1,4]. Diabetes mellitus is usually reported in 75 to 100% of patients with fungal MEO [5,6]. In our study, the patients were diabetic in 86% (n=37) of cases. Other immunosuppressive factors such as chronic renal failure, anti-cancer chemotherapy, hematologic malignancies and acquired immune deficiency syndrome (AIDS) have been reported [7,8]. Otalgia is the most reported symptom with nocturnal exacerbation as described in our study for all patients. Otorrhea is the second frequent symptom in 81% (n=35). Other functional signs can be seen such as trismus, dizziness, tinnitus and fever [6,9]. The fungus makes the infection more invasive which explains the higher number of cranial nerve palsies in patients with Fungal MEO [3,6]. This complication was observed in 16% of our patients (n=7).

The most fungus incriminated in MEO is genus *Aspergillus* with predominance of *A. fumigatus* followed by *A. Flavus* [10,11]. In our study *Aspergillus* MEO was diagnosed in 20 cases including 18 cases of positive cultures and 2 cases with positive *Aspergillus* antigenemia. In addition, our series shows the predominance of *A. flavus species. Aspergillus* secrete several proteolytic enzymes that facilitate tissue invasion [10,11]. Furthermore, Susceptibility testing is helpful in defining the activity spectrum of antifungals and determining the appropriate drug for treatment. All strains of *Aspergillus* isolated were sensitive to voriconazole. Resistance to amphotericin B and caspofungin was reported in 50% (n=9) and 72% (n=13) of our isolates, respectively. The second fungal agent frequently responsible for MEO is Candida yeasts. However, in our series, they represent the most frequent isolate in 49% of cases (n=21).These yeasts produce a biofilm forming a barrier to the host's defence mechanisms and to the passage of antifungals [12]. Mycological examination allows identification of yeasts and antifungal susceptibility testing. In our study, no resistance to antifungals was demonstrated for the *Candida* yeasts. *C. parapsilosis* isolates were intermediate to Micafungin in 80% of cases (n=4).In fact, elevated MICs were founded of echinocandin for *C. parapsilosis*.

In two of our patients, *Geotrichum capitatum (Saprochaete capitata)* was isolated. We didn´t found in literature any cases of MEO caused by this fungus. It is rarely responsible for deep invasive infections in patients with hematological malignancies and transplant subjects [13]. Regarding antifungal susceptibility, the two isolates had a low MICs for amphotericin B and voriconazole (<1µg / ml) and were resistant to fluconazole and caspofungin. In fact, data on its antifungal susceptibilities are limited; however amphotericin B and voriconazole appeared to be the more active drugs. Yenizehirli *et al*. proved in a series of 96 fungal isolates taken from 92 patients suspected of otomycosis, the efficacy of voriconazole [14]. In fact, the mean MICs of voriconazole for *A. fumigatus, A. niger, A. flavus* and *C. albicans* were significantly lower than those of itraconazole for the same pathogens [14]. *Candida* yeasts are frequently sensitive to all triazole derivatives. For MEO due to *Candida*, fluconazole may be prescribed as the first line [14]. Voriconazole is also effective with even greater efficacy than fluconazole [15-17]. The antifungal treatment must be prolonged with a total duration of three to six months [15,17]. In our series, 49% (n=21) of our patients were treated with fluconazole and 51% (n=22) with voriconazole in case of MEO due to *Candida* yeasts and to *Geotrichum/Aspergillus* respectively. Other therapies were used such as surgery and hyperbaric oxygen therapy performed in 5 cases (12%) and 12 cases (28%) respectively. Hyperbaric oxygen therapy promotes healing, angiogenesis, and phagocytosis, by increasing the partial pressure of oxygen [18]. Good outcome was noted in 91% (n=39) of our patients with an average follow-up of 2 years. Fungal MEO is an opportunistic infection increasingly described in the literature but the majority of authors report case studies or series of around ten cases. The strength of our work is the large number of cases collected (n=43), appearing among the largest studies in the literature, which offers reliability to our epidemiological data. However, it should be noted that this study has certain limitations, such as the absence of a standard inter-department protocol which codifies the treatment of this disease which allows reliable comparative statistical studies. Prospective studies are to be carried out in the future in order to resolve the problems of management of this invasive infection.

## Conclusion

Our study proves the predominance of *Candida* yeasts and *Aspergillus* as the fungal agents involved in MEO and reports the first cases due to *Geotrichum capitatum*. Mycological diagnosis allows the identification and antifungal susceptibility testing. Thus, it allows to use the appropriate antifungal treatment and to improve the prognosis of the disease. The antifungal susceptibility testing performed of all our isolates proves the effectiveness of Voriconazole with low MICs. Management of this fungal invasive infection often requires prolonged antifungal treatment and in some cases the need for surgical procedures or hyperbaric oxygen therapy following surgery.

### What is known about this topic

- *Malignant external otitis is a serious and rare infectious emergency with a high mortality and morbidity rate;*
- *It has frequently bacterial origin, but we are currently witnessing the emergence of fungal agents which poses difficulties in diagnosis and management;*
- *Candida yeasts and Aspergillus are the fungal pathogens mainly incriminated*.

### What this study adds

- *Our study allowed the analyse of clinical profile of this fungal infection in Tunisian population;*
- *We report here the first cases of malignant external otitis due to Geotrichum capitatum;*
- *Antifungal susceptibility testing of fungi involved in this infection was performed and proves the effectiveness of Voriconazole for all isolates*.
